# Homoepitaxy of Boron Nitride on Exfoliated Hexagonal Boron Nitride Flakes

**DOI:** 10.1021/acs.nanolett.4c01310

**Published:** 2024-05-31

**Authors:** Johannes Binder, Aleksandra Krystyna Dabrowska, Mateusz Tokarczyk, Adrien Rousseau, Pierre Valvin, Rafal Bozek, Karol Nogajewski, Grzegorz Kowalski, Wojciech Pacuski, Bernard Gil, Guillaume Cassabois, Roman Stepniewski, Andrzej Wysmolek

**Affiliations:** † Faculty of Physics, University of Warsaw, Pasteura 5, 02-093 Warsaw, Poland; ‡ Laboratoire Charles Coulomb, UMR 5221, CNRS-Université de Montpellier, 34095 Montpellier, France; § Institut Universitaire de France, 75231 Paris, France

**Keywords:** homoepitaxy, MOVPE, BN, polytype, rhombohedral, Bernal

## Abstract

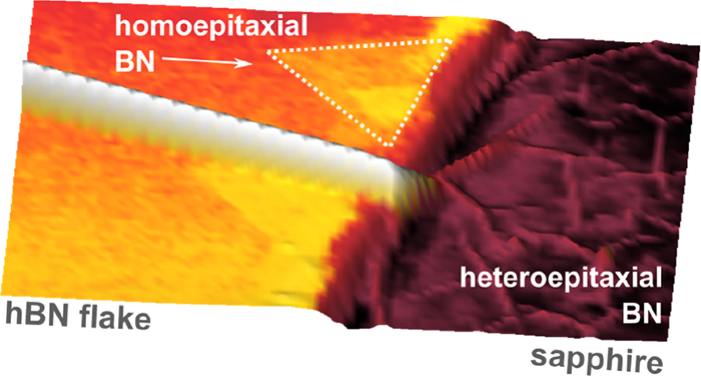

Although large efforts have been made to improve the growth of hexagonal boron nitride (hBN) by heteroepitaxy, the non-native substrates remain a fundamental factor that limits the quality. This problem can be solved by homoepitaxy, which is the growth of hBN on hBN substrates. In this report, we demonstrate the homoepitaxial growth of triangular BN grains on exfoliated hBN flakes by Metal–Organic Vapor Phase Epitaxy and show by atomic force microscopy and photoluminescence that the stacking of these triangular islands can deviate from the AA’ stacking of hBN. We show that the stacking order is enforced by the crystallographic direction of the edge of the exfoliated hBN flakes, with armchair edges allowing for centrosymmetric stacking, whereas zigzag edges lead to the growth of noncentrosymmetric BN polytypes. Our results indicate pathways to grow homoepitaxial BN with tunable layer stacking, which is required to induce piezoelectricity or ferroelectricity.

Boron nitride in its sp^2^-hybridized structure is characterized by a bandgap of about 6 eV and excellent chemical resistance to harsh external conditions.^1,2^ The combination of these properties together with the two-dimensional nature of this compound results in an exceptional versatility of applications, for example as a deep UV light source,^3^ tunnelling barrier,^4,5^ hydrogen barrier,^6,7^ neutron detector,^8^ single-photon emitter,^9,10^ and excellent substrate for growth of other two-dimensional materials.^11−13^ Currently, the best structural, optical and electrical properties for hBN are achieved for bulk material, with dimensions in the millimeter range, obtained from solution-based methods.^1,14^ Epitaxial growth holds great hope for obtaining large-area layers that are more suitable for mass-scale applications. However, the epitaxial layers have not yet achieved the quality of bulk crystals. One of the problems is to find the right substrate for growth. There have been attempts to grow hBN on metallic substrates,^15^ Si/SiO_2_,^16^ SiC,^17^ sapphire^18^ and graphite.^19^ Recently, mechanically polished pyrolytic BN was used as a substrate, resulting in the growth of polycrystalline sp^2^-BN.^20^ Various technologies like Molecular Beam Epitaxy (MBE),^19,21^ Chemical Vapor Deposition (CVD)^15,16,20,22,23^ and Metal–Organic Vapor Phase Epitaxy (MOVPE)^24−29^ were used for epitaxial growth of hBN.

One well-known way to improve the quality of epitaxial layers is to perform homoepitaxy. However, because large bulk hBN crystals are still not available, one has to search for alternative ways of performing homoepitaxy. In this work, we demonstrate a growth process on high-quality hBN flakes mechanically exfoliated from bulk hBN on sapphire. The sapphire substrates with hBN flakes were prepared by means of nondeterministic exfoliation from bulk hBN crystals.

Figure 1 presents an atomic force microscope image (AFM) of the surface of an overgrown exfoliated flake. The most prominent observation after the growth process is the wrinkle formation on the exfoliated hBN flakes, which can be seen as bright yellow lines in the AFM image. These wrinkles are formed during cooling after the growth in agreement with previous reports^30−32^ that show wrinkle formation after annealing at high temperatures. These macroscopic wrinkles do not arise from BN overgrown by MOVPE and appear also for annealing processes performed without BN growth precursors. They intersect at an angle of 120°, demonstrating the excellent structural quality of the exfoliated bulk material. More experimental data on these macroscopic wrinkles can be found in the Supporting Information. Next to the flake one can also see a wrinkle pattern, but with a much smaller mesh size. These microscopic wrinkles appear on the BN layer that grows directly on the sapphire substrate.

**Figure 1:**
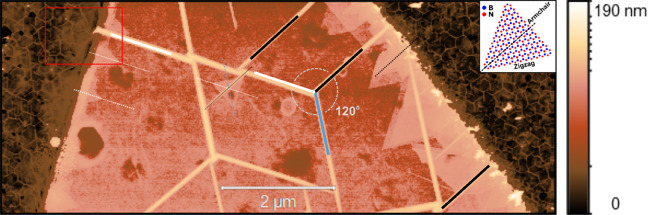
AFM image showing the surface of an exfoliated flake and adjacent regions on sapphire. Large wrinkles are clearly apparent on the flake. The bold black, blue, and white lines correspond to the armchair directions of the flake.^28^ Homoepitaxially grown triangular islands are visible mostly at the edges, but can also be found on the flake. The inset in the upper right corner shows a graphical illustration of a triangular BN island. The edges of the triangles are along the zigzag and the apothem along the armchair directions, in agreement with the orientation of the wrinkles of the underlying flake (the thin dashed and solid bold lines run parallel to each other). The red rectangle indicates the area shown in Figure 5 a).

Since the wrinkle patterns form during the cool down after the growth, they are not interfering with the actual growth taking place at high temperatures. As a sign of homoepitaxial growth, we can clearly see triangular grains that form preferentially at the edges of the exfoliated flake. To prove that these triangular grains are the result of the MOVPE growth, we present AFM images of the surface of a flake before and after the growth process in the Supporting Information. Because of the ideal flatness of the exfoliated flake and the lack of dangling bonds out of plane, there are almost no nucleation sites available that would allow efficient synthesis of BN on top of the flake. However, the edge at the borders of the flake can act as nucleation sites, which is exactly what we observe in Figure 1, as triangular islands form almost exclusively at the edges. On the whole AFM image only one triangular grain is present inside the flake. We also observe that the total amount of material grown on the flake is much lower than the amount of material grown in the same process directly on the surface of sapphire.

To further assess the crystalline orientation of the triangular grains grown on top of the exfoliated flake, we compare the orientation of the triangles to the orientation of the wrinkles that form on the exfoliated hBN flake during the cool down. Earlier reports^30,32^ show that these wrinkles primarily form along the armchair directions of hBN. Therefore, we can directly assess the crystalline orientation of the monocrystalline hBN flake using the wrinkle structure. The inset in the upper right corner of Figure 1 illustrates the orientation of the triangular grain with regard to the wrinkles on the flake. It has been reported that the energetically most preferential form are triangular BN grains with nitrogen terminated zigzag edges.^33−36^ Therefore, in the case of epitaxial growth the armchair direction should run along the apothem of the equilateral triangle, as depicted in the inset of Figure 1. The armchair directions of the wrinkles on the flake are indicated by bold black, white and blue lines in Figure 1. The dashed black, white, and blue lines indicate the same directions, but are shifted and superimposed on top of the triangular grains. A comparison between the dashed lines and the orientation of the apothem of the triangle shows that all the triangular grains, including the triangular grain appearing on the flake, show a perfect epitaxial correlation, indicating homoepitaxial growth of BN on hBN (for more examples see Supporting Information). An important question that arises concerns the polytype of the homoepitaxial BN. In the case of hexagonal boron nitride, AA’ stacking should be observed. However, there are other, noncentrosymmetric polytypes of BN like rhombohedral rBN (ABC stacking)^37,38^ or Bernal bBN (AB stacking),^36,39^ which have been already measured experimentally. The simplest way to distinguish between hexagonal and other polytypes is to assess the orientation of consecutive triangular grains in multilayer BN.^33,36^ For hBN, the second triangular grain should be rotated by 60 degrees. However, for rBN, bBN or AA stacked BN the triangular grains should be aligned parallel to each other. Such multilayer triangular BN islands can be observed close to the right edge of the flake in Figure 1. The stacked triangular grains are oriented with parallel edges, which corresponds to a noncentrosymmetric BN polytype. The observation of a noncentrosymmetric polytype on top of the centrosymmetric hBN flake demonstrates that it is possible to grow homoepitaxial BN heterostructures with different stacking by MOVPE. Such a growth of heterostructures of different polytypes on top of each other can be very interesting for future ferroelectric or piezoelectric applications, but can also be used in terms of bandgap engineering, for example to trap charge carriers in BN-based DUV lighting applications.^40^

Far from the edge of the flake, however, we also observe stacked triangular grains showing the 60 degree rotation typical for AA’ stacking (Figure 2 a). Interestingly, for single triangular grains far from the edges, we observe both possible orientations, as can be seen in the AFM image in Figure 2 b. The parallel, stacked triangular islands in the lower left edge are enforced by the near edge, but the three small triangular grains nucleated on the flake. Two of these small triangular grains have the same orientation as the triangular grains from the edge, but one triangular grain is rotated by 60 degrees. This finding indicates, that for one orientation the triangular grain grows according to the stacking sequence of the flake (AA’), but the other triangular grain is rotated, which indicates a noncentrosymmetric stacking. This finding is in agreement with theoretical predictions that the different stacking sequences AA’, AB, ABC and AB1 (interlayer rotation of 60 degrees and shift so that the layers stack boron on top of boron) are very close in energy.^36,41^ Here, we show that different stacking can be observed even in homoepitaxial growth on the same flake right next to each other.

**Figure 2:**
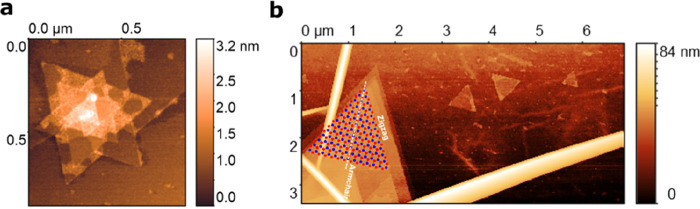
(a) AFM image of triangular islands inside the flake that show a 60 degree rotation between consecutive layers, as expected for the AA’ stacking. (b) AFM image of a stack of parallel triangular grains close to the edge (lower left) and three small triangular grains that nucleated on the flake. Two of these triangular grains are oriented in parallel, but the third triangular grain is rotated by 60 degrees indicating a different stacking.

It has been shown that a deviation from the AA’ stacking leads to changes in the electronic structure of BN.^36,38,39,42^ For the case of Bernal stacking (bBN) the direct and indirect excitons have been found to become quasi-degenerate, with an additional line at around 6.035 eV appearing in the photoluminescence (PL) spectrum.^39^ To corroborate our findings based on AFM, we performed scanning deep-ultraviolet (DUV) PL measurements with submicrometer resolution.^39^Figure 3 a presents an optical image of one of the overgrown hBN flakes. Figure 3 b displays a false-color map of the PL signal integrated in the range between 6.03 and 6.05 eV. For hBN no emission is expected in this range, but for bBN we expect to see an additional line. It can be clearly seen that there is a region with intense emission in this spectral range (typical PL spectra are shown in the Supporting Information). For a noncentrosymmetric crystal we also expect to obtain a second-harmonic generation (SHG) signal. The map in Figure 3 c shows the integrated PL signal in the SHG range. Clearly the most intense signal is observed for the region, which also shows an intense signal that we ascribe to bBN. On the flake, however, no signal is observed in agreement with the AA’, centrosymmetric stacking of the hBN. The comparison with the AFM image (Figure 3 d) shows that there are large triangular grains in this region (for clarity the edges of the triangles are marked with dashed blue lines). We hence can conclude that even a few noncentrosymmetrically stacked BN layers on top of a bulk exfoliated hBN crystal can give rise to a measurable characteristic PL signal of bBN and a strong SHG signal.

**Figure 3:**
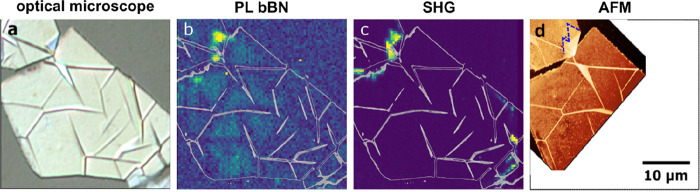
Optical properties of homoepitaxial triangular grains on the exfoliated flake. (a) Optical image of an exfoliated flake after the growth. The wrinkle pattern, as shown in Figure 2, is clearly apparent. (b) False-color map of the PL intensity integrated in the energy range from 6.03 to 6.05 eV, corresponding to bBN. (c) False-color map of the second harmonic generation signal (energy range: 6.15–6.17 eV) (d) AFM image of the sample area. The blue dashed lines indicate the presence of triangular grains. The scale bar shown in (d) applies to all panels.

The results presented above indicate that different polytypes can be grown directly on exfoliated hBN flakes. We further show that the edge of the flake plays an important role in the growth and can enforce whether we obtain a centrosymmetric or noncentrosymmetric stacking of triangular grains. One way to explain this observation is to treat the edge as a linear diffusion barrier that acts as a nucleation site. The accumulation of boron and nitrogen across the diffusion barrier at the edges will lead to faster growth along the edge. Since we observe triangular growth, we can intuitively understand that a preferred growth along the edge will mean that the longest intersections across the triangular grain should be orientated along this edge. In other terms, we do not expect that the triangular grains start to grow from a single point (apex of the triangle) outward but along the diffusion barrier, which is the edge, with the apex oriented inward. Experimental evidence for this thesis is provided in the AFM image in Figure 4 a. The image shows the edge of an exfoliated flake. From the large wrinkles on the flake, one can deduce the crystallographic direction of the flake. The upper edge of the flake is in the zigzag direction, whereas the lower-right edge runs along the armchair direction. Interestingly, for the zigzag terminated edge of the exfoliated flake, we find only triangular grains that are stacked in a noncentrosymmetric way (all triangles are oriented in parallel). However, for the armchair terminated edge of the exfoliated flake, we observe two different orientations as schematically indicated in Figure 4 b. This observation can be understood by assuming epitaxial growth and simple geometrical considerations (Figure 4 c). For the zigzag edge, the longest cross section across the triangle is the base, and there are no equivalent configurations, so a parallel stacking is enforced. However, for the armchair edge of the exfoliated flake we see that there are two equivalent orientations of the triangles, since the longest intersection through the triangle is along the apothem.

**Figure 4:**
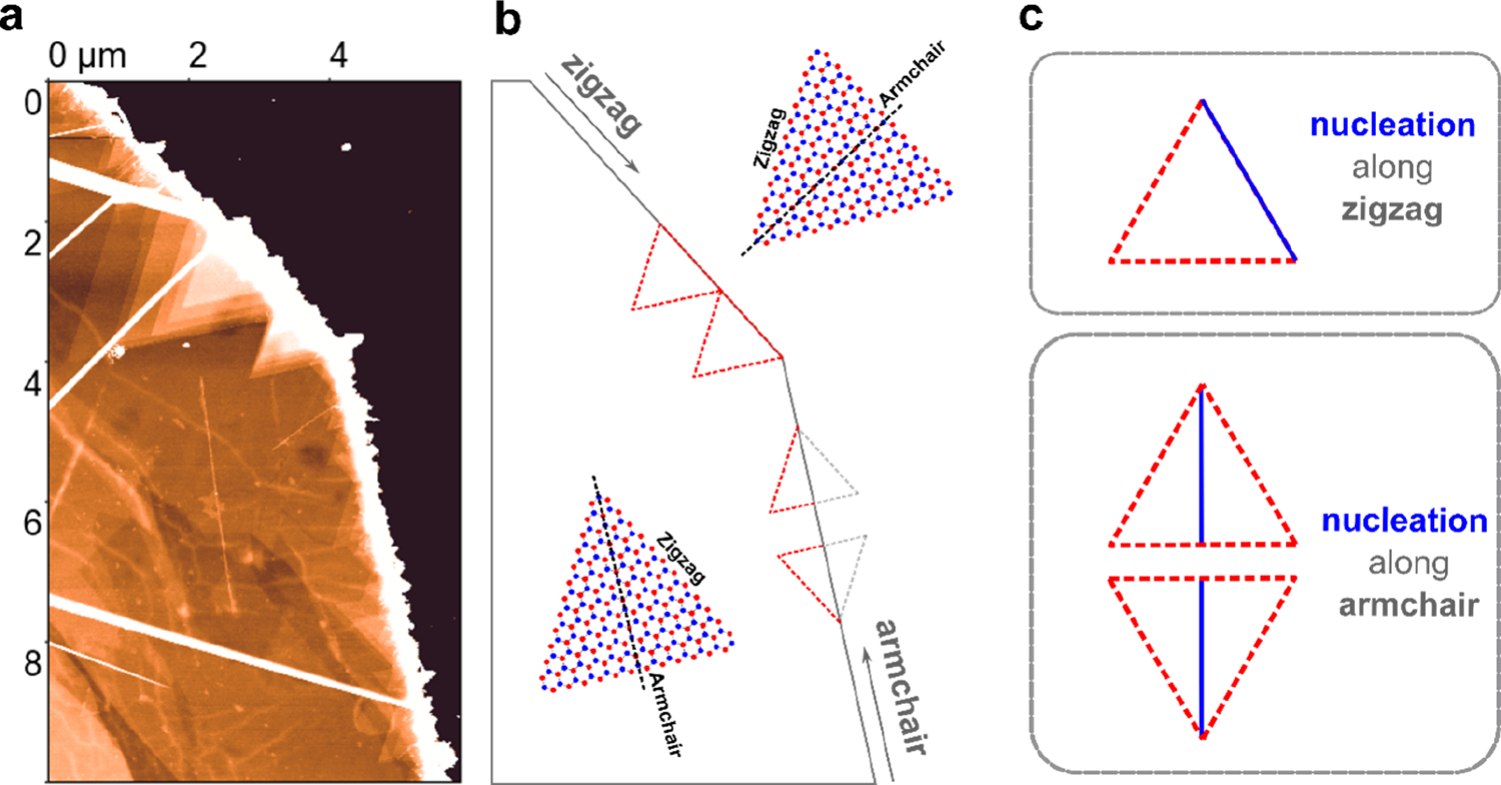
Influence of flake edges on the stacking sequence. (a) AFM image of the surface a flake for which the upper edge runs along the zigzag and the lower right edge along the armchair direction. (b) Schematic drawing of the situation observed in (a). The observed orientation of triangular grains are indicated schematically. At the zigzag terminated edge only triangular grains that are oriented in parallel are observed. For the armchair terminated edge of the exfoliated flake, also triangular grains that are rotated by 60 degrees are observed. (c) Illustration of the nucleation enforced by the edge. For the zigzag direction, the edge enforces the triangular grains to grow from the base of the triangle, whereas for the armchair edge the nucleation parallel to the edge leads to the growth along the apothem of the triangle. The armchair edge allows for centrosymmetric stacking (60 degree rotation) whereas the zigzag edge enforces a noncentrosymmetric stacking (no rotation between consecutive layers).

These considerations indicate that the edges of a flake or any other linear nucleation site can be used to preferentially grow centrosymmetric or noncentrosymmetric polytypes, which may guide the way toward BN homoepitaxy of dedicated polytypes.

On our samples boron nitride is not only grown homoepitaxially on top of the exfoliated flakes in the form of few layer triangular grains, but also on the sapphire substrate in between the flakes with a thickness of about 5 nm. Generally, the surface morphology of the hBN epilayer on the sapphire substrate is dominated by a net of microscaled wrinkles. As in the case of the macroscopic wrinkles on the exfoliated flakes, these smaller wrinkles form during the cooling after the growth process,^28^ as a result of a difference in the thermal expansion coefficients of sapphire and boron nitride.^43−45^

An interesting aspect is the growth mechanism of boron nitride at the interface between the flake and the sapphire substrate. The macroscopic wrinkles on the hBN flake smoothly turn into a mesh of microwrinkles on the sapphire (Figure 5), which clearly shows that the flake and the epitaxial layer are interconnected (for more examples, see Supporting Information). The flake may hence act as a seed for lateral growth, which may be useful to improve the quality of the layer growing in between the flakes.

**Figure 5:**
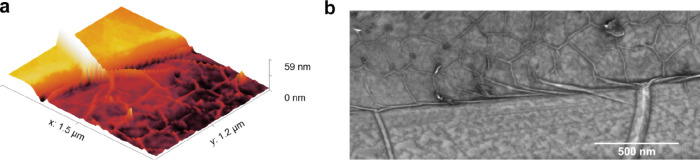
(a) AFM image of the area indicated by the red rectangle in Figure 1. (b) SEM image of an edge of the exfoliated flake after the growth process. The wrinkle pattern clearly shows that the hBN of the flake and the BN that grew on the sapphire are interconnected.

In summary, we have demonstrated homoepitaxy of high-quality BN on mechanically exfoliated hBN flakes. We observe triangular homoepitaxial growth, which preferably occurs at the edges of the flake. Due to the lack of dangling bonds out-of-plane and the atomic flatness of hBN, nucleation is limited on the flat exfoliated flakes, not allowing for efficient homoepitaxial growth. Nucleation occurs preferentially on defects or at the edges of the flakes. We have shown that the crystallographic orientation of the edge of the exfoliated flake can have a strong influence on the polytype of the homoeptiaxially grown triangular grains. Armchair edges are compatible with the AA’ stacking, whereas zigzag edges preferentially lead to noncentrosymmetric stacking. Stacks of multiple homoeptiaxial BN triangular grains observed close to the edges, showed parallel orientation, which indicates the presence of a noncentrosymmetric BN polytype on top of the centrosymmetric (AA’) stacked hBN flake. Deep UV microphotoluminescence measurements revealed characteristic spectra for Bernal BN (AB-stacked) and second harmonic generation, further corroborating the presence of different polytypes in BN homoepitaxy.

The presented results constitute a first step toward homoepitaxy of BN. By carefully manipulating the crystallographic orientation of the nucleation sites it could become possible to control the growth of centrosymmetric hexagonal (AA’ stacked) or noncentrosymmetric polytypes like Bernal BN (AB-stacking) or rhombohedral BN (ABC). This additional degree of freedom could open up a pathway for heterostructures made solely of BN that include piezoelectric or ferroelectric layers.

## Methods

The growth was carried out in an AIXTRON CCS 3 × 2’ MOVPE system. Sapphire pieces (1 × 1 cm) randomly covered with exfoliated hBN flakes were used as a substrate. The growth was conducted in the Continuous Flow Growth (CFG) regime,^21,22^ for which both precursors (ammonia and triethylboron) are injected simultaneously into the reactor. Hydrogen was used as a carrier gas, and the ratio of nitrogen and boron precursor flows was set to 400. The growth temperature was 1300 °C and the pressure was 800 mbar.

The surface morphology of the obtained boron nitride layers was characterized by Scanning Electron Microscopy (SEM) using a FEI Helios NanoLab 600 system and by Atomic Force Microscopy (AFM) with a BRUKER Dimension Icon 6.

The sapphire substrates decorated with hBN flakes were prepared by means of nondeterministic exfoliation from bulk hBN crystals delivered by *hq graphene* and high-quality back-grinding tape from Nitto Denko Corporation. The substrates were sonicated in isopropanol for 10 min and annealed on a hot plate at 200 °C for at least 15 min before exfoliation. The tape was left on top of the sapphire substrates for several hours and then peeled off very slowly to ensure the highest possible yield of hBN flakes transferred from the tape to the substrates.

Deep ultraviolet hyperspectral cryomicroscopy is performed with the setup described in ref (39). Briefly, PL excitation is provided by the fourth harmonic of a continuous-wave (cw) mode-locked Ti:Sa oscillator and it is tunable from 193 to 205 nm with trains of 140 fs pulses at 80 MHz repetition rate. SHG is excited by the second harmonic of the Ti:Sa oscillator. The injection path uses a series of metallic and dichroic mirrors coated for these spectral UV ranges. The exciting laser beam is focused by a Schwarzschild objective located inside the closed-cycle cryostat equipped with CaF_2_ optical windows. The objective numerical aperture is 0.5, resulting in a laser spot size of 200 nm in PL spectroscopy. The sample is mounted on a stacking of piezoelectric steppers and scanners, which is cooled down to 6 K under ultrahigh vacuum (10^–11^ bar). The emission is collected by means of an achromatic optical system comprising a pinhole for confocal filtering. It is then dispersed in a Czerny–Turner spectrometer with 500 mm focal length and a 1200 grooves/mm ruled grating and finally detected by a back-illuminated CCD camera with 13.5 μm pixel size.

## Data Availability

The data that support the findings of this study are available online 10.58132/OJYEIU.

## Supporting Information Available

AFM images of a flake before and after growth, additional AFM/SEM images of triangular grains and wrinkles as well as photoluminescence spectra (PDF)
