# Supplementary material for: Homoepitaxy of Boron Nitride on Exfoliated Hexagonal Boron Nitride Flakes
Source: Nano Lett. 2024 May 31;24(23):6990–6. doi: 10.1021/acs.nanolett.4c01310 (PMC11177313; doi:10.1021/acs.nanolett.4c01310)
Supplement: Supplementary file 1 — nl4c01310_si_001.pdf [file nl4c01310_si_001.pdf]

Supplementary information for

Homoepitaxy of boron nitride on exfoliated hexagonal boron nitride flakes

Johannes Binder[1], Aleksandra Krystyna Dabrowska [1], Mateusz Tokarczyk [1], Adrien Rousseau [2], Pierre Valvin [2], Rafal Bozek [1], Karol Nogajewski [1], Grzegorz Kowalski [1], Wojciech Pacuski [1], Bernard Gil [2], Guillaume Cassabois [2,3], Roman Stepniewski [1], Andrzej Wysmolek [1]*

[1] Faculty of Physics, University of Warsaw, Pasteura 5, 02-093 Warsaw, Poland

[2] Laboratoire Charles Coulomb, UMR 5221, CNRS-Université de Montpellier, 34095 Montpellier, France

[3] Institut Universitaire de France, 75231 Paris, France

Surface morphology of exfoliated h-BN flakes before and after MOVPE growth

To show that the triangular grains discussed in the main text are resulting from the MOVPE growth process, we present atomic force microscopy (AFM) images of the surface morphology of a typical exfoliated flake measured before and after the growth process (Figure S1). The images are taken on the same flake and from the same area, so they can be directly compared. In Fig. S1 a and c (before the growth) no triangular grains are observed. The large wrinkles that are apparent on these images originate from the exfoliation process. After the growth process (Fig. S1 b,d) these initial wrinkles, which originate from the exfoliation, vanish and a new mesh of wrinkles appears, as described in the main text. Moreover one can clearly see (Fig. S1 d) that triangular grains appear at the edges of the exfoliated flake.

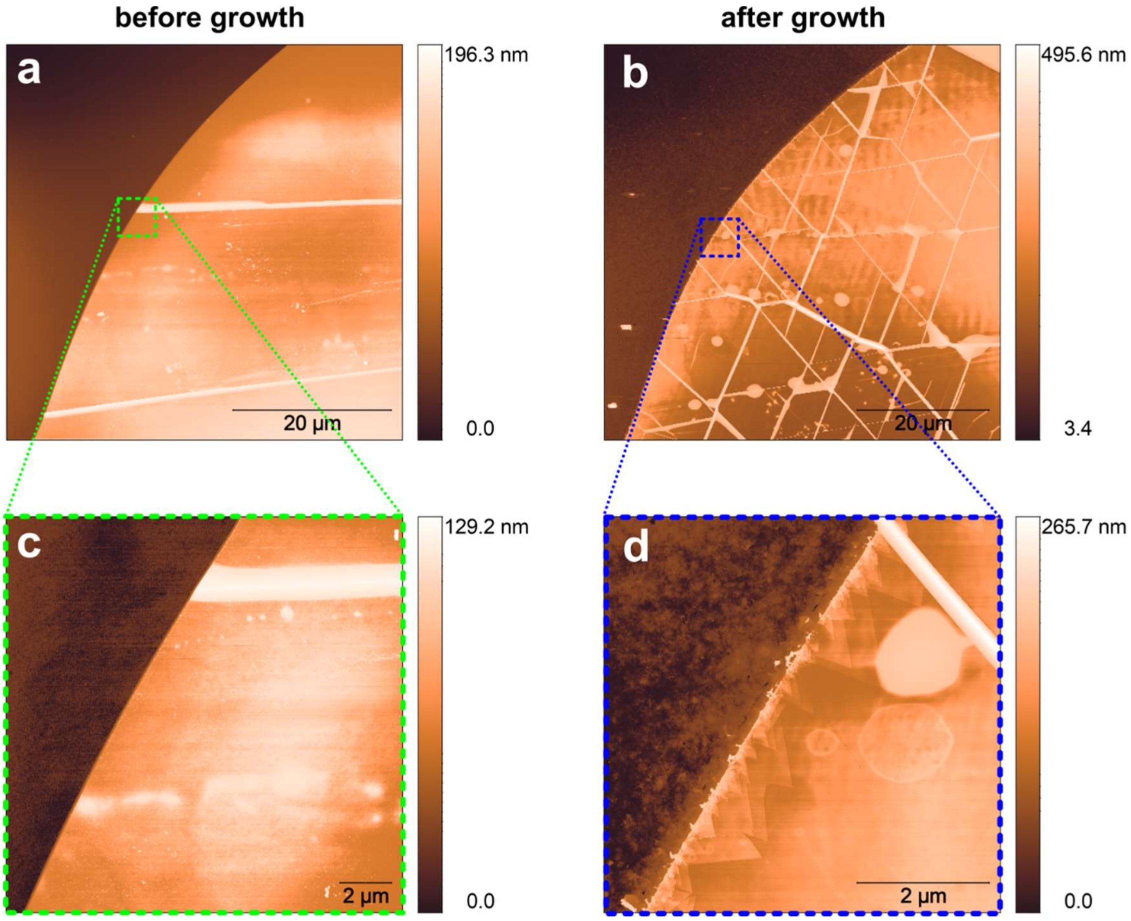

Figure S1: Atomic force microscope (AFM) images of a hBN flake before and after the MOVPE growth process. (a) shows the surface morphology at the edge of the exfoliated hBN flake before growth. No triangular grains are observed, also for a higher resolution image presented in (c). The green dashed square in (a) indicates the area, which is shown in (c). (b) presents the same area of the same flake after growth. One can clearly identify the mesh of large wrinkles and triangular grains at the edge of the flake. (d) shows a higher resulting image of the are indicated by the blue dashed square in (b).

The absence of triangular grains before the growth and their appearance after the growth process clearly shows that the results discussed in the main text can explained by homoepitaxial growth of BN on hBN.

Macroscopic wrinkles and orientation of homoepitaxial triangles

Figure S2 presents scanning electron microscope (SEM) images of overgrown exfoliated hBN flakes. The macroscopic wrinkles that form after cooling on the exfoliated flake as a result of the differences in the thermal expansion of hBN and sapphire are clearly visible.

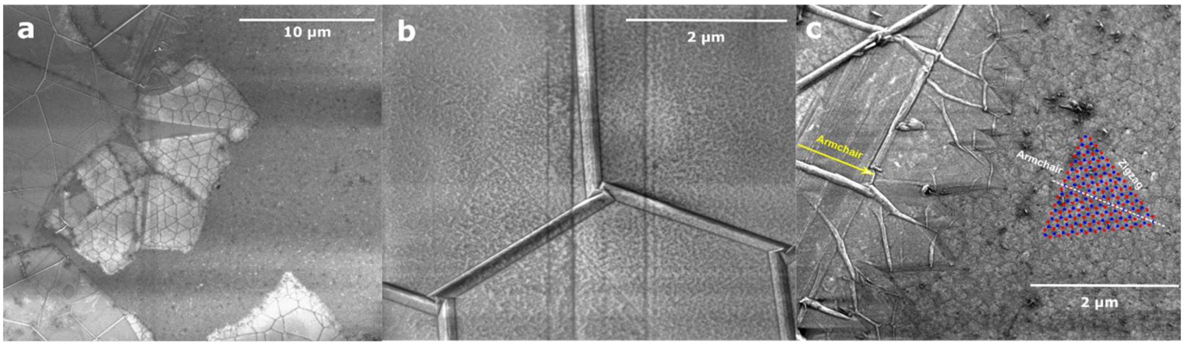

Figure S2: Scanning electron microscope (SEM) images of hBN flakes after the MOVPE growth procedure. (a) Different overgrown flakes and a region where BN grew directly on sapphire. (b) Image of a macroscopic wrinkle appearing on the flake (tilt angle 35 degrees). (c) Interface between a thin hBN flake and sapphire (tilt angle 35 degrees). The macroscopic wrinkle runs parallel to the armchair direction as indicated by the yellow arrow. A schematic triangle with nitrogen-terminated edges indicates the orientation of the triangles that appear at the interface.

An image of such a typical macroscopic wrinkle is shown in Fig. S2 b. These wrinkles are not related to growth and appear also for processes without TEB and NH_3 precursors. Wrinkle formation of this type has been described by Chen at al.[1] and the orientation of the wrinkles has been shown to be along the armchair directions. Figure S2 c shows the interface between a thin exfoliated flake and the sapphire substrate. The macroscopic wrinkles on the flake can be used to assess the direction of the edge of the flake, which in this case is across the zigzag direction. In agreement with the geometrical arguments given in the main text, the zigzag edges result in a noncentrosymmetric stacking, which is observed as a stack of triangles for which the edges are oriented in parallel. This example indicates that the edge acts as a diffusion barrier from both sides. In this case of a thin exfoliated hBN flake this led to the formation of triangles not on top, but as an extension of the flake on the sapphire. A closer look onto the triangles additionally shows a pattern of smaller wrinkles that originates from the BN grown on sapphire and is even visible on top of the triangles. This indicates that the actual mechanism of the overgrowth is complex and depends on the thickness of the flakes.

Figure S3 presents another AFM image that directly illustrates the influence of the crystallographic orientation of the edges of the exfoliated flakes, similar to what is shown in Fig. 4 in the main text.

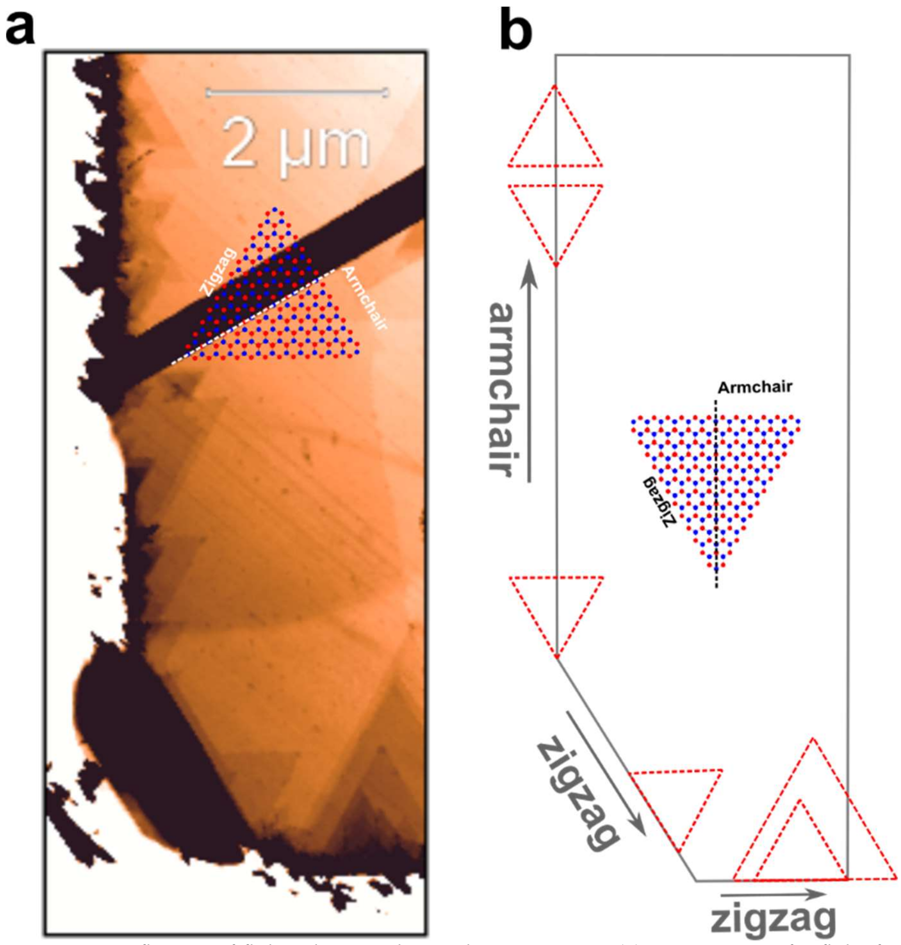

Figure S3. Influence of flake edges on the stacking sequence. (a) AFM image of a flake for which the left edge runs along the armchair and the lower right edge along the zigzag direction. (b) schematic drawing of the situation observed in (a).

In this image one edge is armchair terminated while the other two edges are along the zigzag direction. In agreement with the example presented in Fig. 4 in the main text, the base of the triangles at the zigzag edges are oriented along the zigzag direction whereas the apothem of triangles runs along the armchair direction. Even though the edges are not perfectly sharp, we again observe two orientations in the case of the armchair direction.

Photoluminescence spectra

Figure S4 presents typical photoluminescence spectra from the maps presented in Fig. 3 in the main text.

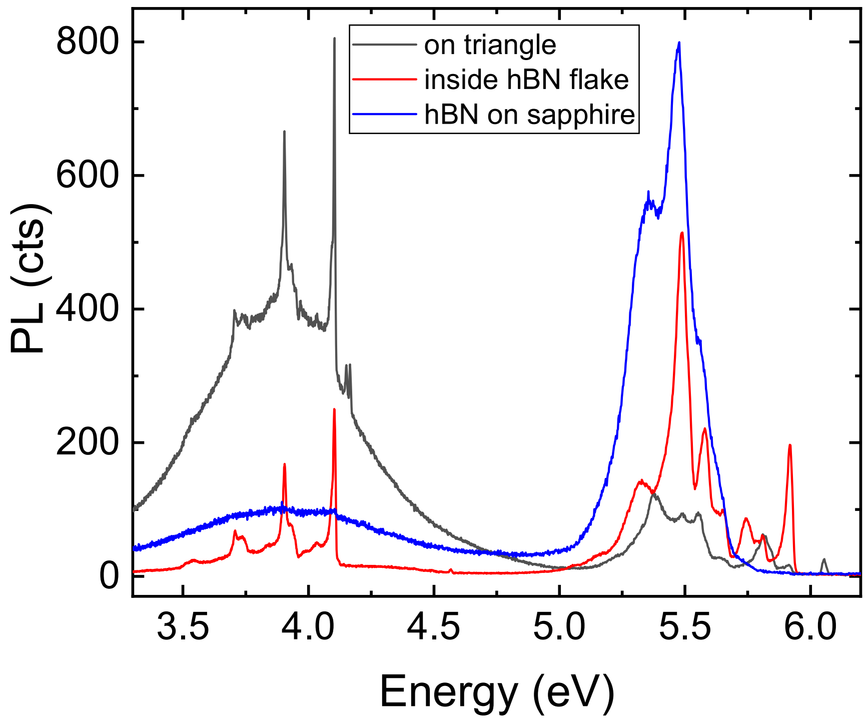

Figure S4. Typical PL spectra of 3 different regions presented in Fig. 3 in the main text. The blue curve was measured next to the exfoliated flake, showing the spectrum of BN grown directly on sapphire. The red spectrum corresponds to a spot on the exfoliated flake far from the edges. Drawn in black is a spectrum taken on the triangle shown in the main text.

The black spectrum shows additional features that are not observed for BN grown on the sapphire substrate next to the flakes and for regions on the exfoliated flakes far from the edges. The black spectrum taken on the triangles show a clear peak in the range 6.03 eV - 6.05 eV together with two distinct bands at a slightly larger energy than the 4.1 eV defect band. These features are indicative of AB-stacked BN (Bernal BN, bBN) as shown in earlier reports [2-3].

References

[1] L. Chen, K. Elibol, H. Cai, C. Jiang, W. Shi, C. Chen, H. S. Wang, X. Wang, X. Mu, C. Li, K. Watanabe, T. Taniguchi, Y. Guo, J. C. Meyer, and H. Wang, "Direct observation of layer-stacking and oriented wrinkles in multilayer hexagonal boron nitride," *2D Materials*, vol. 8, no. 2, p. 024001, 2021.

[2] A. Rousseau, P. Valvin, W. Desrat, L. Xue, J. Li, J. H. Edgar, G. Cassabois, and B. Gil, “Bernal boron nitride crystals identified by deep-ultraviolet cryomicroscopy,” *ACS Nano*, vol. 16, no. 2, pp. 2756–2761, 2022.

[3] A. Rousseau, P. Valvin, C. Elias, L. Xue, J. Li, J. H. Edgar, B. Gil and G. Cassabois, “Stacking-dependent deep level emission in boron nitride,” *Phys. Rev. Materials,* vol. 6, pp. 09400916, 2022
